# Synergistic Effect of Sodium Hypochlorite and Carbon Dioxide Against *Enterococcus faecalis* Biofilm

**DOI:** 10.3390/dj13090417

**Published:** 2025-09-10

**Authors:** Júlia Guerra de Andrade, Ana Flávia Folhas Natali, Caroline Loureiro, Gladiston William Lobo Rodrigues, Ana Paula Fernandes Ribeiro, Rayara Nogueira de Freitas, Renan Jose Barzotti, Laura Cesário Oliveira, Yuri Gabriel Chamorro de Moraes, Natália Amanda Gomes, Antônio Hernandes Chaves-Neto, Frederico Canato Martinho, Rogério de Castilho Jacinto

**Affiliations:** 1 Department of Restorative Dentistry, Araçatuba School of Dentistry, São Paulo State University (UNESP), Araçatuba 16015-050, SP, Brazil; julia.guerra@unesp.br (J.G.d.A.); anaflavia.natali@hotmail.com (A.F.F.N.); caroline.loureiro@unesp.br (C.L.); gladiston.william@unesp.br (G.W.L.R.); apf.ribeiro@unesp.br (A.P.F.R.); laura.cesario@unesp.br (L.C.O.); yuri.chamorro@unesp.br (Y.G.C.d.M.); natalia.a.gomes@unesp.br (N.A.G.); 2 Department of Basic Sciences, Araçatuba School of Dentistry, São Paulo State University (UNESP), Araçatuba 16015-050, SP, Brazil; rayara.nogueira@unesp.br (R.N.d.F.); renan.barzotti@unesp.br (R.J.B.); antonio.hernandes@unesp.br (A.H.C.-N.); 3 Department of Advanced Oral Sciences and Therapeutics, School of Dentistry, University of Maryland, Baltimore, MD 21201, USA; fmartinho@umaryland.edu

**Keywords:** root canal irrigants, sodium hypochlorite, carbon dioxide, bacteria, biofilm

## Abstract

**Objectives:** This study aimed to evaluate whether the addition of pressurized carbon dioxide (PCD) influences the antimicrobial efficacy of 2.5% sodium hypochlorite (NaOCl) against *Enterococcus faecalis* biofilm in root canals and dentinal tubules. **Methods:** Forty extracted human mandibular premolars with single canals were contaminated with *E. faecalis* for 10 days and randomly assigned to four groups (*n* = 10): 2.5% NaOCl, 2.5% NaOCl + CO_2_, sterile saline, and sterile saline + CO_2_. The pH and temperature of the NaOCl solution were measured before and after CO_2_ incorporation. Microbial load was assessed by CFU counts before and after irrigation, and in dentin samples from the cervical, middle, and apical thirds. Oxidative stress was evaluated via lipid peroxidation (TBARS), protein carbonyl content, and total protein quantification. Biofilm metabolic activity was analyzed using the XTT reduction assay. Data were analyzed using one-way ANOVA on ranks and two-way repeated measures ANOVA (α = 0.05), a very large effect size (Cohen’s *d*) ≈ 1.756 was assumed. **Results:** All irrigation protocols significantly reduced bacterial load (*p* < 0.05). Both NaOCl groups outperformed the saline controls (*p* = 0.009). The addition of CO_2_ to NaOCl slightly enhanced disinfection in the main canal but did not improve antimicrobial action in dentinal tubules. CO_2_ incorporation reduced the pH of NaOCl from ~13.4 to 7.4 and slightly increased the temperature, making the solution more chemically reactive. However, both oxidative stress markers and the XTT assay showed that the combination with CO_2_ impaired the antimicrobial effectiveness of NaOCl. **Conclusions:** Despite the improvement in bacterial reduction in the root canal lumen, the combination of PCD with NaOCl failed to enhance intratubular disinfection and reduced the oxidative damage and metabolic inactivation of the biofilm. CO_2_ pressurization appears to limit the antimicrobial action of NaOCl.

## 1. Introduction

Effective root canal decontamination remains one of the main challenges in endodontics, particularly when dealing with resistant microorganisms such as *Enterococcus faecalis* [1]. The persistence of microbial communities and their virulence factors within the root canal system is a leading cause of endodontic treatment failure [2]. Although these secondary endodontic infections are polymicrobial in nature, they are predominantly composed of Gram-positive facultative anaerobes, notably *E. faecalis* [3,4,5]. This species is frequently implicated in endodontic failure due to its ability to survive in hostile environments—such as its tolerance to highly alkaline conditions—and to form resilient biofilms in anatomically complex areas, including dentinal tubules [6,7,8,9,10]. In addition to its biofilm-forming capacity and coaggregation with other species, *E. faecalis* expresses multiple virulence factors that contribute to its resistance to conventional endodontic disinfection protocols [11,12]. Therefore, the eradication of mature biofilms remains a significant challenge in endodontic therapy.

Sodium hypochlorite (NaOCl) is the most widely used endodontic irrigant due to its broad-spectrum antimicrobial activity and ability to dissolve organic tissues [13,14]. Various concentrations of NaOCl, ranging from 0.5% to 6%, have been tested for root canal disinfection [15,16]. Although the antimicrobial effect of NaOCl increases with concentration, higher concentrations also lead to increased cytotoxicity in periapical tissues [17,18,19]. Therefore, achieving effective cleaning, lubrication, and disinfection within the root canal system is critical to the success of endodontic therapy [20]. Additionally, it has been reported that lowering the pH of NaOCl to a range between 6.0 and 7.5 may enhance its antimicrobial efficacy. This is because when dissolved in water, chlorine undergoes hydrolysis and predominantly forms hypochlorous acid (HOCl)—the most active disinfecting species—which is favored at lower pH levels. As a result, NaOCl becomes chemically more reactive and effective under mildly acidic conditions [21].

To improve the efficacy of NaOCl, several enhancement strategies have been investigated, including temperature elevation [22], ultrasonic activation [23,24], and negative pressure irrigation systems [25]. Moreover, clinical scenarios such as endodontic treatment in teeth with immature apices [26], or procedures based on revascularization protocols—which rely on the regenerative potential of the periapical tissues—still require further clarification regarding irrigation protocols and potential adjuvants. These cases are particularly sensitive due to the increased risk of irrigant extrusion beyond the apex [27].

Among potential adjuncts, pressurized carbon dioxide (PCD) has emerged as a promising candidate due to its recognized antimicrobial properties. PCD has shown substantial efficacy in inactivating a variety of pathogenic organisms in both aqueous and non-aqueous environments [28,29,30]. Its favorable physicochemical properties—such as low cost, non-toxicity, low viscosity, and zero surface tension—facilitate rapid penetration into porous and complex structures [28]. Studies have indicated that combining PCD with NaOCl enhances bacterial inactivation in certain contexts, such as seawater disinfection [28], possibly due to pH reduction that increases the proportion of HOCl [31].

In addition to microbial inactivation, it is essential to evaluate whether CO_2_ enhances the oxidative stress exerted by NaOCl on bacterial biofilms. The innate immune system commonly employs oxidative stress as a defense mechanism, involving the production of reactive oxygen species (ROS) such as superoxide (O_2_^−^) and hydroxyl radicals (OH^−^), as well as non-radical oxidants like hydrogen peroxide (H_2_O_2_) and reactive nitrogen/chlorine species including nitric oxide (NO) and HOCl [32,33]. These oxidants contribute not only to microbial destruction but also to tissue damage and inflammation. Thus, understanding how oxidative mechanisms are modulated during irrigation is essential to balance microbial elimination with biocompatibility [32,33].

Although various strategies have been proposed to enhance the antimicrobial action of NaOCl [22,23,24,25], no studies to date have evaluated the effect of combining NaOCl with PCD for root canal disinfection. In particular, the impact of this combination on oxidative damage to microbial structures—such as lipid peroxidation and protein oxidation—remains entirely unexplored in the endodontic literature. Given the growing interest in improving irrigant performance through physical–chemical modifications, this gap represents a relevant area for investigation. Therefore, the present in vitro study aimed to evaluate the efficacy of combining PCD with NaOCl against *E. faecalis* biofilm in root canals and dentinal tubules, using microbiological culture methods and biochemical assays to assess lipid peroxidation and protein oxidation. The null hypothesis was that the addition of PCD to NaOCl would not result in a significant difference in CFU reduction or oxidative damage when compared to NaOCl alone.

## 2. Materials and Methods

### 2.1. Sample Selection and Preparation

This study was approved by the Research Ethics Committee of the School of Dentistry, Araçatuba—UNESP (CAAE: 34692620.8.0000.5420, approved on 23 September 2020). Forty human mandibular premolars recently extracted for periodontal or orthodontic reasons were selected. Teeth with anatomical alterations, previous treatments, or structural damage—such as fractures, open apices, root curvature > 10°, or restorations—were excluded. All the extracted teeth were stored in 0.1% thymol solution and refrigerated at 2–4 °C from the time of extraction until use (approximately 6 months).

Residual periodontal tissues were removed with periodontal curettes. The crowns were sectioned transversely with a water-cooled diamond disk (American Burrs, Palhoça, Santa Catarina, Brazil), and the root segments were standardized to 16 mm in length. Working length was determined by inserting a size 10 K-file (Dentsply Sirona, Ballaigues, Switzerland) into the canal until its tip was visible at the apical foramen under magnification [34]. Root canals were instrumented using rotary files up to size 40.04 (MK Life, Porto Alegre, RS, Brazil) according to the manufacturer’s speed and torque specifications. During instrumentation, 1 mL of 2.5% NaOCl (Rioquímica, São José do Rio Preto, SP, Brazil) was used for irrigation after each file.

The irrigation protocol for cleaning dentinal tubule entrances was adapted from Ferraz et al. (2001) [35]. All the samples were subjected to an ultrasonic bath for 10 min in 17% EDTA (Biodinâmica, Ibiporã, PR, Brazil), followed by 10 min in 5.25% NaOCl (Apothimed, Araçatuba, SP, Brazil) to remove the smear layer. Irrigants were neutralized with 5 mL of 5% sodium thiosulfate (Merck KGaA, Darmstadt, Germany). Each tooth was immersed in approximately 700 mL of Brain Heart Infusion (BHI) broth (Kasvi, Pinhais, Paraná, Brazil) and ultrasonically agitated for 1 min to promote penetration of the culture medium into canal irregularities. The samples were then autoclaved for 30 min at 121 °C and incubated at 37 °C for 48 h to confirm sterilization efficacy.

### 2.2. Cultivation of E. faecalis and Specimen Contamination

The contamination protocol was adapted from Carvalho et al. (2019) [36]. A pure culture of *E. faecalis* (ATCC 29212, ATCC, Manassas, VA, USA) was grown in BHI broth at 37 °C for 24 h. The culture was transferred to fresh BHI and incubated overnight at 37 °C to reach exponential growth. The cellular suspension was adjusted in a spectrophotometer (BioTek Instruments, Winooski, VT, USA) at 600 OD to achieve a turbidity of 1.5 × 10^−8^ colony-forming units (CFU/mL), equivalent to 0.5 McFarland standard [37], to be used for biofilm formation.

Aliquots of 800 μL were introduced into 2 mL Eppendorf tubes containing the specimens, followed by sequential centrifugation at 1400, 2000, 3600, and 5600× *g* in double 5 min cycles. The inoculum was renewed after each cycle. After eight cycles, the samples were transferred to new 2 mL tubes with sterile BHI broth, vortexed (Phoenix Luferco, Araraquara, Brazil), and incubated at 37 °C for 24 h. For 10 days, the specimens were maintained in sterile BHI broth, with centrifugation (3600× *g* for 5 min at 25 °C) on alternate days to enhance bacterial penetration into the root canal system and for a mature biofilm. The culture medium was renewed every 48 h on the centrifugation days. All the steps were performed aseptically in a laminar flow cabinet (Veco Bioseg 12 Ltd.a, Campinas, Brazil). On the tenth day, the samples were removed, excess medium discarded, and external root surfaces cleaned with sterile gauze.

### 2.3. Experimental Groups

Sample size calculation was based on previous studies [37,38], indicating 10 teeth per group. A very large effect size (Cohen’s *d*) ≈ 1.756 was assumed, with a significance level (α) of 0.05 and a statistical power (1–β) of 0.80 [39]. After completing the sample size determination (n), the teeth were placed in 2 mL Eppendorf tubes, randomly numbered, and blindly allocated by a single operator into four experimental groups according to the irrigation protocol employed:

- *The 2.5% NaOCl group*: Root canals were irrigated and manually agitated with a #15 K-file using 15 mL of 2.5% NaOCl, 5 mL of 17% EDTA, and a final 5 mL of 2.5% NaOCl for 20 s each. Subsequently, 1 mL of 5% sodium thiosulfate was used for 1 min to inactivate NaOCl. A total of 20 mL of 2.5% NaOCl was used for irrigation [36,40].
- *The 2.5% NaOCl + CO_2_ group*: Root canals were irrigated and manually agitated with a #15 K-file using 15 mL of 2.5% NaOCl + CO_2_ (NaOCl was carbonated using a jet aeration device -Sodastream Industries LTD, Kfar Saba, Israel), 5 mL of 17% EDTA, and a final 5 mL of 2.5% NaOCl + CO_2_ for 20 s each. Subsequently, 1 mL of 5% sodium thiosulfate was used for 1 min to inactivate NaOCl. A total of 20 mL of 2.5% NaOCl + CO_2_ was used for irrigation [36,40].
- *Sterile saline group*: Root canals were irrigated and manually agitated with a #15 K-file using 15 mL of sterile saline solution, 5 mL of 17% EDTA, and a final 5 mL of sterile saline solution for 20 s each. Subsequently, 1 mL of 5% sodium thiosulfate was used for 1 min. A total of 20 mL of sterile saline solution was used for irrigation [36,40].
- *Sterile saline + CO_2_ group*: Root canals were irrigated and manually agitated with a #15 K-file using 15 mL of sterile saline solution + CO_2_ using the jet aeration system, 5 mL of 17% EDTA, and a final 5 mL of sterile saline solution + CO_2_ solution for 20 s each. Subsequently, 1 mL of 5% sodium thiosulfate was used for 1 min. A total of 20 mL of sterile saline solution + CO_2_ was used for irrigation [36,40].

After the contamination period, the specimens were mounted on a sterile aluminum platform. The apical foramen of each sample was sealed with a fast-setting epoxy resin to prevent apical leakage and to simulate a closed canal system. All the irrigation procedures were carried out using 30-G NaviTip needles (Ultradent Products Inc., Indaiatuba, Brazil), positioned 1 mm short of the working length.

CO_2_ was incorporated into the 2.5% NaOCl + CO_2_ and Sterile Saline + CO_2_ groups using a jet aeration system (Sodastream Industries LTD, Kfar Saba, Israel). A CO_2_ gas cylinder was connected to the device, and the gas was infused into 800 mL of 2.5% NaOCl by pressing the release button 10 times to ensure consistent CO_2_ saturation. The same procedure was applied to 800 mL of sterile saline to maintain consistency across experimental groups.

### 2.4. Microbiological Collection

The sampling method used in this study was based on the protocol described by Yamamoto et al. (2021) [37]. Briefly, microbiological samples were collected from all the root canals before (S1) and after (S2) irrigation using three sterile paper points (Dentsply Maillefer). Each point remained in the canal for one minute before being transferred to 2 mL Eppendorf tubes containing 1 mL of Ringer’s solution (Sigma-Aldrich, St. Louis, MI, USA). The tubes were vortexed for 30 s. The samples were homogenized and serially diluted to 10^−4^ (S1) and 10^−1^ (S2). Each dilution was plated on BHI agar and incubated at 37 °C for 48 h. Colony-forming units (CFU/mL) were then counted.

### 2.5. Dentine Samples

**Figure 1:**
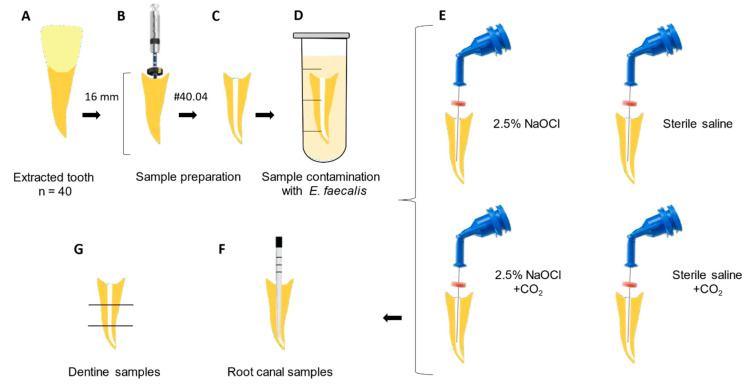
Study design: (**A**) Extracted tooth used in experimental groups. (**B**) Sample preparation: standardization of the root canal length (16 mm) and endodontic treatment with Rotatory Files #40/04. (**C**) Root canal filled with BHI solution. (**D**) Sample contamination with *E. faecalis* for 10 days. (**E**) Experimental groups. (**F**) Root canal sample collection after irrigation protocols. (**G**) Dentine sample collection.

Each root was sectioned into three segments: cervical, middle, and apical thirds (Figure 1). Dentin debris was collected from the canal walls using sterile, incrementally sized diamond-tipped conical burs [4137 (ISO 025), KG Sorensen, Serra, ES, Brazil] mounted on a low-speed electric handpiece (Dentsply Sirona). The collected dentin shavings were individually stored in 2 mL Eppendorf tubes containing Ringer’s solution [41], plated on BHI agar, and incubated at 37 °C for 48 h for CFU quantification.

### 2.6. pH Measurement

The pH of the 2.5% NaOCl solution was measured in its pure form and after the addition of CO_2_ at different pressure levels (1 to 10 gas injections) using a digital pH meter (model PHS3BW, Bel Engineering, Monza, Italy) calibrated at 25 °C. The measurements were taken at 30 and 60 s after each CO_2_ injection.

### 2.7. Assessment of Oxidative Damage Products

The oxidative damage assay was based on Rodrigues et al. (2025) [42]. The *E. faecalis* strain was reactivated on BHI agar and transferred to BHI broth, then incubated at 37 °C for 7 h. After incubation, the bacterial suspension was adjusted using a spectrophotometer to reach a turbidity equivalent to the McFarland 0.5 standard (1.5 × 10^8^ CFU/mL). A total of 45 mL of the inoculum was transferred to 50 mL Falcon tubes and centrifuged at 10,000× *g* rpm at 4 °C for 10 min to form a pellet, and then the supernatant was discarded. The centrifugation process was repeated 12 times to increase the concentration of the bacterial pellet. After the final centrifugation, sterile saline was added to resuspend the pellet, and the suspension was aliquoted into 60 microcentrifuge tubes (1.5 mL).

The tubes were centrifuged again to concentrate the pellet. The supernatant was discarded, and each group (*n* = 10) received the treatment protocol corresponding to its experimental group. The pellets were washed with 2 mL of 0.9% NaCl to remove residual treatment agents. Homogenates were prepared in 50 mmol/L sodium phosphate buffer (pH 7.4) containing 0.2% (*v*/*v*) Triton X-100 and 2 mmol/L phenylmethylsulfonyl fluoride (PMSF). The samples were sonicated for 10 s at 100% amplitude and centrifuged at 10,000× *g* rpm for 10 min at 4 °C. The supernatants were collected for analysis of thiobarbituric acid-reactive substances (TBARS) and protein quantification.

### 2.8. Metabolic Activity Assessment

The metabolic activity of the biofilms was evaluated using the XTT reduction assay (2,3-bis [2-methoxy-4-nitro-5-sulfophenyl]-5-[(phenylamino)carbonyl]-2H-tetrazolium; Sigma-Aldrich, St. Louis, MO, USA), following a protocol adapted from Jacob et al. (2020) [43]. The *E. faecalis* strain (ATCC 29212) was initially cultured on BHI agar (Kasvi, Paraná, Brazil) for 24 h at 37 °C. Subsequently, it was transferred to liquid BHI broth and incubated at 37 °C until reaching the exponential growth phase, with the optical density adjusted to OD_600_ ≈ 0.08 (~1.5 × 10^8^ CFU/mL). The standardized inoculum (200 µL) was distributed into 96-well plates and statically incubated at 37 °C for 72 h to allow biofilm formation. After this period, the biofilms were subjected to the treatments described for each experimental group. CO_2_ activation was performed as previously described, using a solution pre-saturated with the gas under standardized conditions. Preliminary tests defined the final protocol: direct application of 25 µL of the solution for 30 s. After treatment, wells were washed twice with PBS buffer (pH 7.0).

The XTT assay was conducted using a fresh solution composed of XTT (150 mg/L) and phenazine methosulfate (PMS, 10 mg/L) mixed in a 1:1 (*v*/*v*) ratio. Each well received 200 µL of the solution, followed by incubation for 3 h at 37 °C in the dark under mild orbital shaking (120 rpm). After incubation, the 200 µL was transferred to a new plate, and absorbance was measured at 490 nm. The absorbance values were corrected by the negative controls (without biofilm) and used as a relative measure of metabolic activity, considering uniform adhesion to the well bottoms. The experiments were performed in duplicate (*n* = 10 per group) and repeated in two independent assays to ensure reproducibility.

### 2.9. Statistical Analysis

The CFU data were statistically analyzed using Sigma Plot 12.0 for Windows (Systat Software Inc., San Jose, CA, USA). Two-way repeated measures ANOVA followed by the Student–Newman–Keuls post hoc test was employed for intergroup comparisons and analysis across different sampling time points. A one-way analysis of variance on ranks was conducted to evaluate the percentage reduction in CFU counts. Intragroup comparisons among the cervical, middle, and apical thirds were also conducted using two-way repeated measures ANOVA followed by the Student–Newman–Keuls post hoc test. One-way ANOVA followed by Tukey’s post hoc test was applied for the analysis of the other assays. A significance level of 5% (*p* < 0.05) was adopted for all the statistical tests.

## 3. Results

Table 1 presents the intergroup comparison of *E. faecalis* counts before and after the irrigation protocols, along with the percentage of bacterial reduction. Cultivable bacteria were detected in all the baseline samples (S1, 40/40). All the irrigation protocols significantly reduced the bacterial load (*p* < 0.05). Irrigation with 2.5% NaOCl demonstrated superior antibacterial efficacy compared to sterile saline (*p* = 0.001), regardless of CO_2_ supplementation. Notably, the addition of CO_2_ enhanced the decontamination potential of 2.5% NaOCl. However, incorporating CO_2_ into the sterile saline solution did not lead to a significant improvement in bacterial reduction (*p* > 0.05).

Table 2 presents the results of the inter- and intra-group analyses. The 2.5% NaOCl + CO_2_ group demonstrated statistically significant differences among all root thirds, with the highest reduction in CFU/mL observed in the cervical third. In contrast, no significant differences were detected among the cervical, middle, and apical thirds in either of the sterile saline groups.

### 3.1. Results of pH Measurement

**Figure 2:**
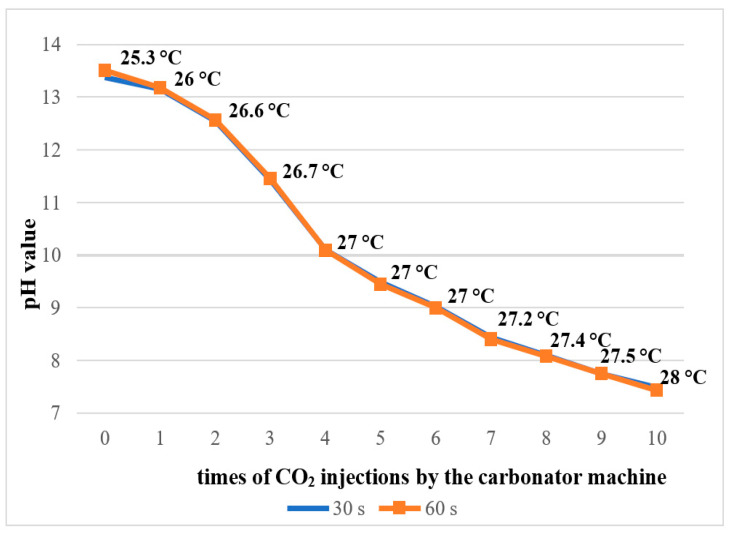
pH measurement and temperature of 2.5% NaOCl solution during the addition of CO_2_ by the carbonator machine.

The pure 2.5% NaOCl solution exhibited an alkaline pH ranging from 13.39 to 13.51 at 25 °C. As the number of CO_2_ gas injections increased, the pH progressively decreased, reaching a final value of 7.43 after 10 injections (Figure 2). A corresponding increase in solution temperature was also observed, from 25.3 °C in the untreated solution to 28 °C following the 10 CO_2_ injections.

### 3.2. Oxidative Damage

**Figure 3:**
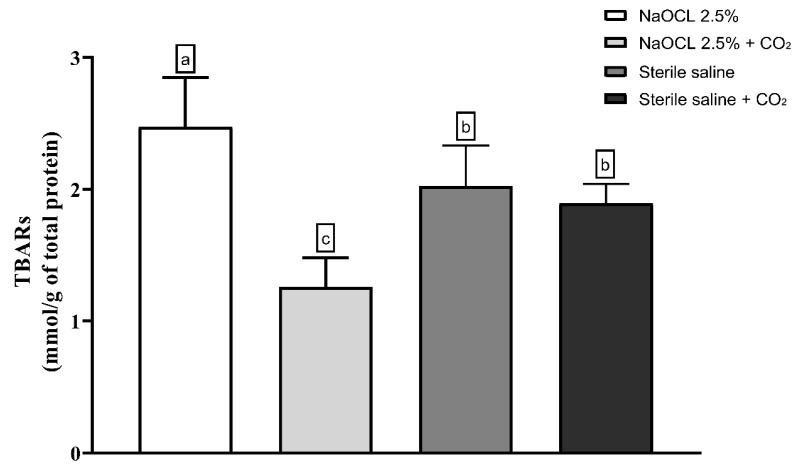
Mean and Standard Deviation of TBARS concentrations in *E. faecalis* cells after treatment with different irrigating solutions: NaOCl 2.5%, NaOCl 2.5% + CO_2_, saline solution, and saline solution + CO_2_. Different lowercase letters indicate statistically significant differences among the groups (*p* < 0.05).

**Figure 4:**
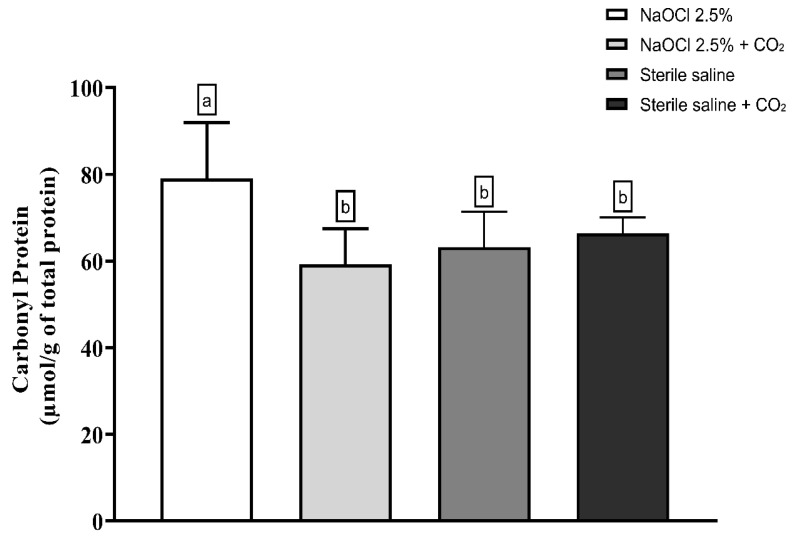
Mean and Standard Deviation of carbonyl protein concentrations in *E. faecalis* cells after treatment with different irrigating solutions: NaOCl 2.5%, NaOCl 2.5% + CO_2_, saline solution, and saline solution + CO_2_. Different lowercase letters indicate statistically significant differences among the groups (*p* < 0.05).

**Figure 5:**
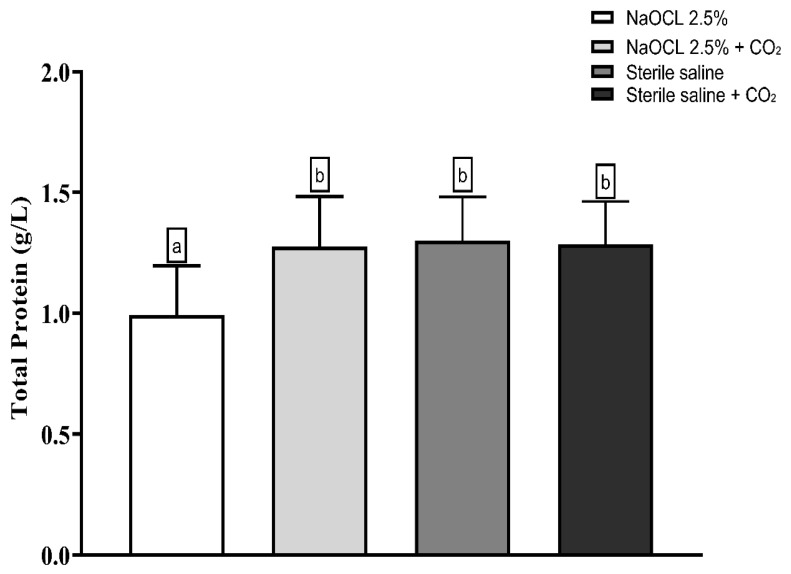
Mean and Standard Deviation of total protein concentrations in *E. faecalis* cells after treatment with different irrigating solutions: NaOCl 2.5%, NaOCl 2.5% + CO_2_, saline solution, and saline solution + CO_2_. Different lowercase letters indicate statistically significant differences among the groups (*p* < 0.05).

Lipid peroxidation was evaluated using the thiobarbituric acid-reactive substances (TBARS) assay. The results showed that the addition of pressurized CO_2_ reduced the ability of 2.5% NaOCl to induce lipid damage (Figure 3). Similarly, protein oxidation—measured by the concentration of carbonylated proteins—was also attenuated when NaOCl was combined with CO_2_ (Figure 4). Furthermore, analysis of total protein levels revealed that only NaOCl without CO_2_ was effective in significantly reducing protein content (Figure 5), suggesting a greater impact on bacterial cell viability.

### 3.3. Evaluation of Metabolic Activity

**Figure 6:**
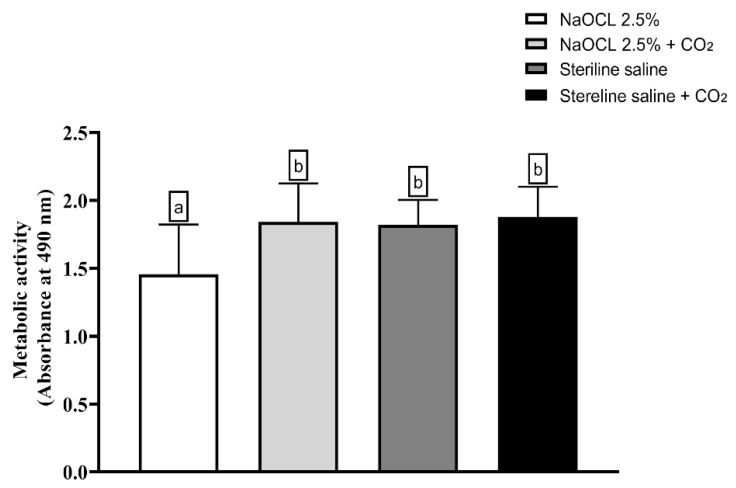
Analysis of the metabolic activity of *Enterococcus faecalis* ATCC 29212 monospecies biofilms exposed to the different experimental groups described in the graph. Different lowercase letters indicate statistically significant differences between groups (one-way ANOVA, Tukey’s post hoc test; *p* < 0.05).

The analysis of the metabolic activity of *E. faecalis* biofilms is shown in Figure 6 demonstrated that only the group treated with NaOCl showed a statistically significant reduction in cell viability compared to the control (*p* < 0.05). The other groups—saline solution, saline solution with CO_2_, NaOCl with CO_2_, and control—did not show significant differences among themselves, indicating that the addition of CO_2_ compromised the antimicrobial activity of NaOCl.

## 4. Discussion

This study investigated the potential of combining PCD with NaOCl for disinfecting root canals and dentinal tubules contaminated with *E. faecalis*. Although NaOCl remains the gold standard in endodontic irrigation due to its broad-spectrum antimicrobial activity and tissue-dissolving properties, strategies to enhance its penetration and efficacy—particularly within dentinal tubules—continue to pose a significant challenge.

Our results showed that 2.5% NaOCl significantly reduced the bacterial load in the main canal space compared to saline controls, which aligns with previous studies emphasizing its superior efficacy [44,45]. The addition of CO_2_ led to a modest improvement in disinfection within the main canal, potentially due to reduced surface tension and pH modification that may enhance hypochlorous acid availability. However, this synergistic effect was not observed in the dentinal tubules, where the anatomical complexity and limited irrigant penetration remain critical obstacles [46].

One plausible explanation for the lack of enhanced intratubular efficacy is the absence of irrigant activation. In industrial settings, PCD typically requires mechanical agitation to promote gas solubilization and enhance penetration into target substrates [47]. In endodontics, passive ultrasonic irrigation (PUI) and other activation techniques have been shown to significantly enhance irrigant distribution and disrupt bacterial biofilms [48,49]. To better reflect clinical conditions and explore the full potential of this strategy, future studies could include irrigant activation protocols combined with CO_2_ and assess their efficacy against a wider range of oral pathogens.

The biochemical analysis revealed that the addition of CO_2_ to NaOCl resulted in significantly reduced oxidative damage to lipids and proteins, suggesting interference with the formation of ROS such as HOCl. This finding deserves further attention, as the acidification of NaOCl can influence the chemical equilibrium between chlorine species. While a slight pH reduction (~pH 6–7.5) may favor the predominance of HOCl over hypochlorite ions (OCl^−^), excessive acidification, as observed with CO_2_ saturation to pH 7.4, may destabilize the oxidative activity of NaOCl and compromise its antimicrobial action [21,46]. Thus, future investigations should assess controlled acidification strategies that balance antimicrobial potency with chemical stability.

Another important aspect concerns the design of the oxidative stress assays. In this study, biochemical analyses were conducted using bacterial pellets in suspension rather than dentin-adhered biofilms, which may limit the translational relevance of the findings. Although this method enables standardized quantification of oxidative damage, it does not accurately reproduce the protective architecture and microenvironment of biofilms residing within dentinal tubules. However, this approach offers important advantages, including experimental reproducibility, precise control of treatment conditions, and the ability to quantify oxidative damage without interference from dentin matrix components. It also enables the isolation of the direct effects of irrigants on bacterial components, helping to elucidate fundamental mechanisms of action. As a preliminary screening tool, this model provides valuable insights that can guide the design of more complex in situ experiments. Notably, the metabolic activity and cellular fragility of the *E. faecalis* biofilm observed in the XTT assay were consistent with the oxidative damage findings. Future studies should employ dentin-adhered biofilm models alongside advanced imaging or spectroscopic techniques to more accurately assess oxidative stress in anatomically complex systems.

In summary, while the addition of CO_2_ to NaOCl presents a conceptually promising approach, the findings of this study suggest that under non-activated conditions, this combination does not improve disinfection in dentinal tubules and may even reduce the chemical aggressiveness and antimicrobial effectiveness of NaOCl. This insight reinforces the notion that the uncritical adoption of new irrigation adjuncts can lead to unintended antagonistic interactions, ultimately diminishing treatment outcomes. Therefore, the use of adjuncts such as PCD must be guided by a comprehensive understanding of their physicochemical interactions with conventional irrigants and the specific conditions required for their optimal performance. A more rigorous control of pH, application of irrigant activation, and clinically relevant oxidative stress assessment models are essential next steps.

## 5. Conclusions

Within the limitations of this in vitro study, the combination of PCD with 2.5% NaOCl resulted in a modest improvement in bacterial reduction within the root canal lumen but did not enhance antimicrobial activity in dentinal tubules. Furthermore, the addition of CO_2_ significantly attenuated oxidative damage to lipids and proteins and reduced biofilm metabolic inactivation—indicating a marked decrease in the overall bactericidal efficacy of NaOCl. Although conceptually promising, this approach requires further refinement, particularly regarding pH modulation, chemical stability, and delivery dynamics. A clinically relevant implication of these findings is that the association of substances with NaOCl—especially when applied without proper activation—may limit their therapeutic potential.

## Figures and Tables

**Table 1:** Mean (±Standard Deviation) of CFU/mL counts (log10) before and after irrigation protocols (IP).

Irrigation Protocol	Before IP	After IP
2.5% NaOCl	6.5 ^Aa^	1.2 ^Ba^
	(±0.5)	(±1.2)
2.5% NaOCl + CO_2_	6.3 ^Aa^	0.4 ^Bb^
	(±0.4)	(±0.7)
Sterile saline	6.7 ^Aa^	5.0 ^Bc^
	(±0.4)	(±0.2)
Sterile saline + CO_2_	6.9 ^Aa^	4.9 ^Bc^
	(±0.3)	(±0.2)

Uppercase letters indicate comparisons within the same group before and after irrigation (*p* < 0.05). Lowercase letters indicate comparisons between different groups using distinct irrigation protocols at the same time point (*p* < 0.05).

**Table 2:** Mean (±Standard Deviation) of CFU/mL counts (log10) after irrigation protocols (IP) in the different thirds.

Thirds	Irrigation Protocol			
	2.5% NaOCl	2.5% NaOCl + CO_2_	Sterile Saline	Sterile Saline + CO_2_
Cervical	0.8 ^Aa^	0.2 ^Aa^	3.9 ^Ba^	4.1 ^Ba^
	(±1.3)	(±0.5)	(±0.6)	(±0.6)
Medium	0.7 ^Aa^	1.0 ^Ab^	4.2 ^Ba^	3.9 ^Ba^
	(±1.6)	(±1.4)	(±0.4)	(±0.9)
Apical	2.7 ^Ab^	3.5 ^Ac^	4.5 ^Ba^	4.5 ^Ba^
	(±1.8)	(±0.6)	(±0.3)	(±0.5)

Uppercase letters indicate comparison within the different groups using different irrigation protocols. (*p* < 0.05). Lowercase letters indicate comparison within the different thirds for each irrigation protocol (*p* < 0.05).

## Data Availability

The original contributions presented in this study are included in the article. Further inquiries can be directed to the corresponding author.
